# Supplementary material for: Case Study: An Evaluation of Detection Dog Generalization to a Large Quantity of an Unknown Explosive in the Field
Source: Animals (Basel). 2021 May 8;11(5):1341. doi: 10.3390/ani11051341 (PMC8151250; doi:10.3390/ani11051341)
Supplement: Supplementary file 1 [file animals-11-01341-s001.zip › Data description.docx]

**Data description**

As part of the supplementary materials, we included all the raw data for all three studies. The data for each study is in a separate sheet withing the xlsx document.

Study 1- The data for study 1 is in the sheet titled “Study One”. Dog Team responses are coded as zeros and ones. Ones represents alerts and zeros no alerts to each sample or bag. Each column shows dog team response to each bag or sample.

Study 2- Data for study two is divided in two sheets. The sheet titled “Study 2 30g-subsample” contains the data for the generalization test with the 30 g subsample of the confiscated explosive. The sheet titled “Study 2 30g-training sample” contains the control test with the 30 g of the training sample. Ones mean an alert and zeros a no response. FA indicates if a false alert during a trial. The “Target” column indicates if the dog alerted to the target odor within a trial.

Study 3- Sheets with named as “Experiment three” and “Experiment 3” contain the data of study 3. It is the same data just organized in a different format. Both sheets show dogs alert to the 13 kg confiscated explosive during the different trials after training with the 30 g subsample. Ones means an alert and zero a no response.
